# VLDL Induced Modulation of Nitric Oxide Signalling and Cell Redox Homeostasis in HUVEC

**DOI:** 10.1155/2017/2697364

**Published:** 2017-09-20

**Authors:** Maria Chiara Magnifico, Roxana Elena Oberkersch, Azzurra Mollo, Luca Giambelli, Yasmine Grooten, Paolo Sarti, Graciela Cristina Calabrese, Marzia Arese

**Affiliations:** ^1^Department of Biochemical Sciences, Sapienza University of Rome, Rome, Italy; ^2^Universidad de Buenos Aires, Facultad de Farmacia y Bioquímica, Departamento de Ciencias Biológicas, Cátedra de Biología Celular y Molecular, Buenos Aires, Argentina; ^3^Blood Transfusion Service and Hematology, Umberto I Hospital, Rome, Italy; ^4^Department of Analytical Chemistry, Applied Chemometrics and Molecular Modelling, Vrije Universiteit Brussel, Brussels, Belgium

## Abstract

High levels of circulating lipoprotein constitute a risk factor for cardiovascular diseases, and in this context, the specific role of the very-low-density lipoproteins (VLDL) is poorly understood. The response of human umbilical vein endothelial cells (HUVEC) to VLDL exposure was studied, especially focusing on the pathways involved in alteration of redox homeostasis and nitric oxide (NO) bioavailability. The results obtained by the analysis of the expression level of genes implicated in the NO metabolism and oxidative stress response indicated a strong activation of inducible NO synthase (iNOS) upon 24 h exposure to VLDL, particularly if these have been preventively oxidised. Simultaneously, both mRNA and protein expression of endothelial NO synthase (eNOS) were decreased and its phosphorylation pattern, at the key residues Tyr495 and Ser1177, strongly suggested the occurrence of the eNOS uncoupling. The results are consistent with the observed increased production of nitrites and nitrates (NOx), reactive oxygen species (ROS), 3-nitrotyrosine (3-NT), and, at mitochondrial level, a deficit in mitochondrial O_2_ consumption. Altogether, these data suggest that the VLDL, particularly if oxidised, when allowed to persist in contact with endothelial cells, strongly alter NO bioavailability, affecting redox homeostasis and mitochondrial function.

## 1. Introduction

Atherosclerosis, one of the principal causes of morbidity and mortality in occidental countries, is a disease affecting large and medium-sized muscular arteries, characterised by endothelial dysfunction and vascular inflammation [1–3]. Dyslipidaemia, and in particular, the increased concentration of cholesterol esters and low-density lipoproteins (LDL) at the arterial intima surface, has long been recognised as a major proatherogenic factor [4] potentially more dangerous for the vessels upon increasing the lipid oxidation level [5–8]. Not only the LDL but also the very-low-density lipoproteins (VLDL), the physiological precursors of the LDL, might share the responsibility of the onset and builds up of the atherosclerotic plaques [9]. The implication in the atherosclerotic lesion of VLDL can be indirect, since the more elevated the VLDL concentration, the higher the LDL accumulation, but also direct due to the straight molecular interactions with the arterial cell components, membranes, and receptors [10, 11].

Evidence has been collected suggesting that, as reported for the LDL [5], also the oxidation of VLDL triggers a cascade of proatherogenic and proinflammatory cell responses [12]. These include lipoprotein retention in the arterial wall and recruitment of macrophages at the vessel level; under these conditions, foam cells are formed and eventually the atherosclerotic plaque builds up [13, 14].

Interestingly, while the role of LDL and their redox state in the atherogenesis have been both intensively studied [15–18], the experimental evidence supporting the direct involvement of VLDL, native or oxidized, in the onset and development of atherosclerosis is still limited.

Previous reports have shown an increased production of reactive oxygen species (ROS) by mitochondria, accumulation of mitochondrial DNA damage, and progressive respiratory chain dysfunction associated with atherosclerosis [19–23]. A growing body of evidence suggests that alterations of the nitric oxide (NO) synthesis may be involved with this disease [18, 24, 25].

As a gaseous cell messenger, NO regulates several pathways both in prokaryotes and in eukaryotes [18, 26–29], including the modulation of cell energetic through the interaction with electron transport chain proteins [30–32]. Very relevant to cardiovascular diseases, the NO generated by endothelial cells plays a crucial role in the blood pressure control via relaxation of the vessel muscular smooth muscle cells [33].

Studies on knockout mice demonstrated that NO produced at low (nM) concentration by the constitutive NO synthases (eNOS and nNOS) exerts vasculo-protective effects, whereas higher NO concentration levels (*μ*M), as those synthesized by the inducible NOS (iNOS), are more often associated with oxi/nitrosative stress of vessels and tissues [34, 35].

Moreover, it has been observed that under condition favoring oxidative stress and inflammation, the impairment of the physiological activity of the eNOS may occur, leading to the so called “uncoupling state” characterized by the production of superoxide O_2_^−•^ instead of NO [36–38].

The present work was aimed at characterising the response of vascular endothelial cells in culture to a time-persistent (24 h) administration of VLDL as purified from human blood sera (native), or oxidised, focusing on the NO metabolism, the ROS production, and the changes of the primary mitochondrial function [39]. Human umbilical vein endothelial cells (HUVEC) have been taken as a model of vascular endothelium, and within the simplification of a monoculture, it allowed a detailed biochemical characterisation of eNOS biosynthesis and function, pointing to a key role played by this enzyme in the early redox unbalance associated to the onset of atherosclerosis.

## 2. Materials and Methods

### 2.1. Cell Culture

Human umbilical vein endothelial cells (HUVEC, ATCC CRL-1730) were cultured on 0.2% (*w/v*) gelatin-precoated flasks or multiwell plates (Falcon, BD Biosciences, USA) and grown at 37°C, 5% CO_2_, 95% air in F-12K medium containing 1.26 g/L glucose, 0.1 mg/mL heparin, 0.03 mg/mL endothelial cell growth supplement (ECGS), 10% fetal bovine serum (FBS), 100 U/mL penicillin, 100 *μ*g/mL streptomycin, and 1.5 g/L sodium bicarbonate.

The day before the experiments, cells were depleted of serum for 4 h and thereafter incubated in serum-free medium for 24 h with native VLDL (n-VLDL) or oxidised VLDL (ox-VLDL), at the concentration of 75 or 140 *μ*g protein mL^−1^, the former assayed only in a subset of experiments.

Controls were carried out in the absence of VLDL, under otherwise identical conditions. When necessary, HUVEC were harvested by trypsinization and centrifugation (900 ×g) and carefully suspended in the working medium, at suitable density (see text). None of the treatments affected cellular viability of HUVEC, as determined by cell counts, morphology, and trypan blue exclusion analysis.

Cell lysis was performed by CelLytic™ MT Cell Lysis Reagent (Sigma-Aldrich, USA) in the presence of the Protease Inhibitor Cocktail (Sigma-Aldrich, USA); protein content was determined according to bicinchoninic acid (BCA) assay (Sigma-Aldrich, USA), and citrate synthase activity, considered as representative of the mitochondrial mass, was measured as described [40].

### 2.2. VLDL Isolation and Ethics Statement

n-VLDL (density: 0.95–1.006 kg/L) were isolated from human sera by preparative ultracentrifugation following the procedure described by Redgrave et al. [41]. Serum samples were collected from typically 40–50 healthy volunteers of age comprised between 20 and 40 years (gender equally represented) after 12 h fasting, in order to minimize the biological variability among the preparations. The presence of chylomicrons in fresh sera was eliminated centrifuging for 30 min at 15,000 rpm. Then, n-VLDL were obtained by ultracentrifugation at 105,000 ×g, for 18 h at 15°C in a 1.006 kg/L density solution. After ultracentrifugation, the supernatant from the top of each tube was carefully aspirated and pooled. n-VLDL were dialysed against 0.25 mM ethylenediaminetetraacetic acid (EDTA) in phosphate-buffered saline (PBS), using a membrane (molecular weight cut-off: 6–8 kDa) at 4°C overnight. All VLDL preparations were filtered through a sterile 0.45 *μ*m filter and, after the evaluation of protein concentration by the BCA (~500 *μ*g/mL), were stored at −70°C until used. The purity of isolated VLDL was checked on agarose gel and sodium dodecyl sulphate polyacrylamide gel electrophoresis (SDS-PAGE), staining with a lipid-specific dye (Sudan Black B) and a protein-specific dye (Coomassie Blue R 250), respectively.

n-VLDL with high degree of purity were obtained as outcome by its apolipoprotein composition determined by SDS-PAGE (Supplementary Figure 1(a) available online at https://doi.org/10.1155/2017/2697364) and characteristic mobility (Supplementary Figure 1(b)) [42, 43]. Human sera were collected anonymously by the Immunohematology Laboratory, Policlinico Umberto I, Sapienza Università di Roma. The procedure described has been approved by the Ethics Committee of Sapienza University (Prot. number 3610-2015).

### 2.3. VLDL Oxidation

VLDL oxidation was carried out according to Guha and Gursky [44] using Cu^2+^ ions (CuSO_4_). Briefly, a solution of purified n-VLDL, at the concentration 0.2–0.4 mg mL^−1^ in EDTA-free PBS, was incubated with CuSO_4_, 40 *μ*M for 6 h; the reaction was then stopped using 1 mM EDTA. Conjugated dienes and thiobarbituric acid-reactive substances (TBARS) were quantified in order to monitor lipid peroxidation [45, 46] (Supplementary Figure 1(c) and (d), resp.).

### 2.4. Real-Time Polymerase Chain Reaction (PCR)

After HUVEC incubation, for 24 h, with n-VLDL or ox-VLDL (140 *μ*g mL^−1^), total RNA was extracted using the Nucleo Spin® RNA isolation kit (Macherey-Nagel, Germany), according to the manufacturer's instructions. Total RNA, 1 *μ*g, was used for the reverse transcription reaction using RT^2^ First Strand Kit (Qiagen, Germany). The cDNA obtained was hybridised to the Human Nitric Oxide Signalling Pathway RT^2^ Profiler PCR Array (Qiagen, Germany).

This array contains oligonucleotide primers matching 84 genes whose expression is controlled by or involved in the signalling of NO, superoxide metabolism, and response to oxidative stress (see Table 1). The array includes also genes that are known to be induced or repressed by NO and whose finding therefore can be used as indicator for the activation of NO cell pathways.

### 2.5. Western Blot

After HUVEC treatments, cells (1 × 10^6^) were lysed with CelLytic MT Reagent (Sigma-Aldrich, USA) in the presence of Protease Inhibitor Cocktail (Sigma-Aldrich, USA). The proteins were separated on 10% sodium dodecyl sulphate-polyacrylamide gel electrophoresis (SDS-PAGE) and transferred on nitrocellulose membranes (Whatman, GE Healthcare, UK), 1 h at 100 mA. After 2 h blocking (PBS with 0.1% Tween 20 and 3% BSA), the membranes were incubated overnight at 4°C with primary mouse monoclonal anti-phospho-Ser1177 and anti-phospho-Thr495 antibodies (BD Transduction Laboratories, USA). Thereafter, the membranes were stripped and reprobed for monoclonal purified mouse anti-eNOS antibodies (BD Transduction Laboratories, USA); *α*-tubulin was used as the reference. The enhanced chemiluminescence (ECL) Horseradish Peroxidase (HRP) anti-mouse secondary antibody (Jackson, Baltimore, PA, USA) was incubated 1 h at 25°C, and chemiluminescence was determined (Amersham, GE Healthcare, UK). Densitometric analysis was carried out by the ChemiDoc™ MP Image Analysis Software (Bio-Rad, USA).

### 2.6. ROS Quantification

Cell ROS generation was assessed by using the fluorescent probe 2′,7′-dichlorodihydrofluorescein diacetate (DCFDA, Sigma-Aldrich, USA), with modifications respect to the protocol generally adopted for adherent cells.

Briefly, HUVEC (70–75% confluent) were incubated with VLDL for 24 h as already described, then trypsinized, and pelleted at 900 ×g for 5 min at 20°C. Cell pellets were resuspended in Hank's buffer at the density 1 × 10^5^ cells mL^−1^ and plated in 24-well (black) plates.

Immediately after the addition of 10 *μ*M DCFDA, the relative fluorescence emission was followed kinetically at 520 nm for 60 min (VICTOR™ Multilabel Counter, PerkinElmer, USA). The value of ROS production is taken at 30 min.

### 2.7. Nitrate/Nitrite Determination

The accumulation of nitrate/nitrite (NOx) in the culture medium was assessed by using the fluorescent probe 2,3-diaminonaphthalene (DAN) (Fluorometric Assay Kit, Cayman Chemical, USA).

After 24 h incubation of HUVEC (~2.5 × 10^5^ cell/mL) with n-VLDL or ox-VLDL, the culture supernatant was centrifuged at 4°C, 1000 ×g for 10 min and the NOx content was measured by adding DAN according to the manufacturer's instructions and using a Fluorescence Plate Reader VICTOR Multilabel Counter (PerkinElmer, USA).

### 2.8. 3-Nitrotyrosine Quantification

The level of 3-nitrotyrosine- (3-NT-) modified proteins was used as marker of protein damage induced in HUVEC by peroxynitrite and quantified using nitrotyrosine ELISA kit (Abcam ab113848, UK).

After treatments with n-VLDL or ox-VLDL at different concentration (75, 140 *μ*g/mL), HUVEC were trypsinized, pelleted at 500 ×g for 10 min, and washed twice using PBS buffer. Then, cells (~1 × 10^6^) were suspended in sample extraction buffer and incubated on ice for 20 min. After centrifugation at 12,000 ×g 4°C for 20 min, the 3-NT levels were determined colorimetrically according to the manufacturer's instructions.

### 2.9. O_2_ Consumption Measurements

HUVEC incubated with n-VLDL or ox-VLDL (140 *μ*g/mL) were harvested and resuspended in the oxygraph medium consisting of 3 mM MgCl_2_ × 6 H_2_O, 10 mM KH_2_PO_4_, 20 mM HEPES, 1 g/L BSA, 110 mM mannitol, 0.5 mM EGTA, and pH 7.1. Cell density and viability were determined by trypan blue exclusion test. Respiration was assayed as previously described [47, 48] using a high-resolution respirometer (Oxygraph-2k; Oroboros Instruments) equipped with two 1.5 mL chambers with thermostats; data were collected and analysed using the built in software DatLab 4.

Cells (1 × 10^6^) were added to the oxygraph chamber containing the medium, and the system let equilibrate for 5 min. Cell plasma membrane was permeabilised to reducing substrates and effectors with digitonin (see text). The optimal digitonin concentration, 2.7 *μ*g/mL, and the incubation time, 10 minutes, were fixed according to [49].

The contribution to cell respiration of any respiratory complex was evaluated according to Kuznetsov et al. with minor modifications [50]. Briefly, 10 min after the addition of digitonin, 8.8 mM pyruvate and 4.4 mM malate were added and the resting complex I-supported respiration was recorded (state 4); ADP, 2 mM, was then added to induce the maximal mitochondrial respiration (state 3), followed by rotenone, 0.5 *μ*M, to specifically inhibit complex I; succinate, 10 mM, was added to induce complex II-supported respiration, and antimycin, 5 *μ*M, was added to inhibit complex III; finally, complex IV-dependent respiration was activated by 2 mM ascorbate and 0.5 mM N,N,N′,N′-tetramethyl-p-phenylenediamine (TMPD).

### 2.10. Citrate Synthase

HUVEC were harvested (1 × 10^6^), lysed, and centrifuged at 13,000 ×g for 10 min. Cell lysates were assayed for citrate synthase activity [40] and for total protein content. HUVEC, used as controls or after incubation with n-VLDL or ox-VLDL, displayed the same protein content (~0.2 mg/mL) and citrate synthase activity (~0.18 *μ*mol/min/1 × 10^6^ cells) (not shown).

### 2.11. Statistical Analysis

Data are the mean ± standard deviation (SD) of at least three independent biological experiments (as specified in the figure legends), each repeated in three technical replicates. For statistical analysis, one-way analysis of variance (ANOVA), followed by Bonferroni-Holm post hoc test, was used for multiple comparisons. *P* values indicated in figures were considered statistically significant by ANOVA. In Table 1, the *P* values for all genes are shown; when followed by ^∗^, *P* values were considered significant by ANOVA.

## 3. Results

The transcriptional activity of HUVEC exposed to 140 *μ*g/mL n-VLDL or ox-VLDL was investigated by targeting genes related to NO metabolism and to oxidative stress. The genes included in the screening have been grouped in functional classes (Table 1), and the mRNA production of each gene has been reported as the relative to that of untreated cells (see Materials and Methods).

### 3.1. eNOS and iNOS Genes

**Figure 1:**
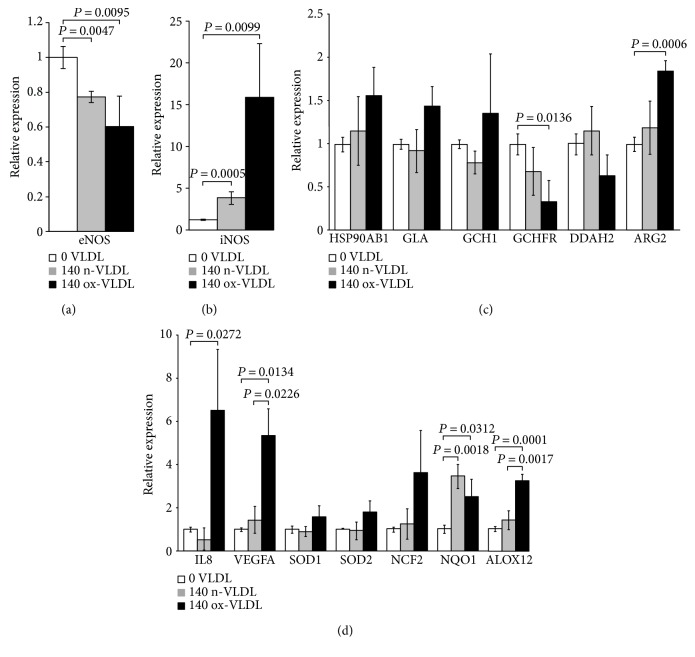
Gene expression changes induced by VLDL. HUVEC were incubated 24 h with n-VLDL (grey) or ox-VLDL (black), both 140 *μ*g/mL. cDNA were assayed by RT-PCR (Human Nitric Oxide Signalling Pathway RT^2^ Profiler PCR Array). The relative expression of the genes of interest is shown. (a) Endothelial nitric oxide synthase (eNOS). (b) Inducible nitric oxide synthase (iNOS). (c) Genes involved in the regulation of eNOS activity. (i) HSP90AB1: heat shock protein 90 kDa *α* (cytosolic), class B member 1; (ii) GLA: galactosidase *α*; (iii) GCH1: GTP cyclohydrolase 1; (iv) GCHFR: GTP cyclohydrolase I feedback regulator; (v) DDAH2: dimethylarginine dimethylaminohydrolase 2; (vi) ARG2: arginase, type II. (d) Genes induced by nitric oxide and involved in superoxide metabolism/oxidative stress response. (i) IL8: interleukin 8; (ii) VEGFA: vascular endothelial growth factor A; (iii) SOD1: superoxide dismutase 1, soluble; (iv) SOD2: superoxide dismutase 2, mitochondrial; (v) NCF2: neutrophil cytosolic factor 2; (vi) NQO1: NAD(P)H dehydrogenase, quinone 1; (vii) ALOX12: arachidonate 12-lipoxygenase, 12S type. Relative expression calculated after *β*-actin normalisation versus control cells. Data ± SD; *n*. of biological experiments = 3. *P* values were considered statistically significant by ANOVA.

As shown in Figures 1(a) and 1(b) following cell incubation with n-VLDL, the eNOS mRNA expression is slightly downregulated by ~0.25-fold, whereas the iNOS is upregulated by ~2.5-fold. When cells were exposed to ox-VLDL, the eNOS mRNA expression was more clearly downregulated (~0.4-fold) while the iNOS increased largely by approximately 15-fold.

### 3.2. Other NO-Related Genes

This group includes genes involved in the stability and functional regulation of the NOS enzyme. Following cell incubation with ox-VLDL, the HSP90AB1, GLA, GCH1, and ARG2 genes are upregulated, whereas the DDAH2 and the GCHFR genes are downregulated (see Figure 1(c)).

Changes of the expression levels of this selection of genes are very small when induced by n-VLDL, varying by a larger extent if VLDL were preliminarily oxidised (see Table 1).

### 3.3. Inflammation-Related Genes

Along with the iNOS mRNA, also the VEGFA-, IL8-, and ALOX12-encoding genes are upregulated by ox-VLDL, pointing to the activation of inflammation pathways (Figure 1(d)). The expression of endothelial genes involved in the NADPH oxidase biosynthesis and function, namely, the NCF2 and NQO1, also appears upregulated together with the SOD1 and SOD2 mRNA, the latter increased by ~1.8-fold (see Table 1 and Figure 1(d)). In this frame, the 24 h cell incubation with n-VLDL is clearly less effective.

### 3.4. eNOS Expression and Uncoupling

**Figure 2:**
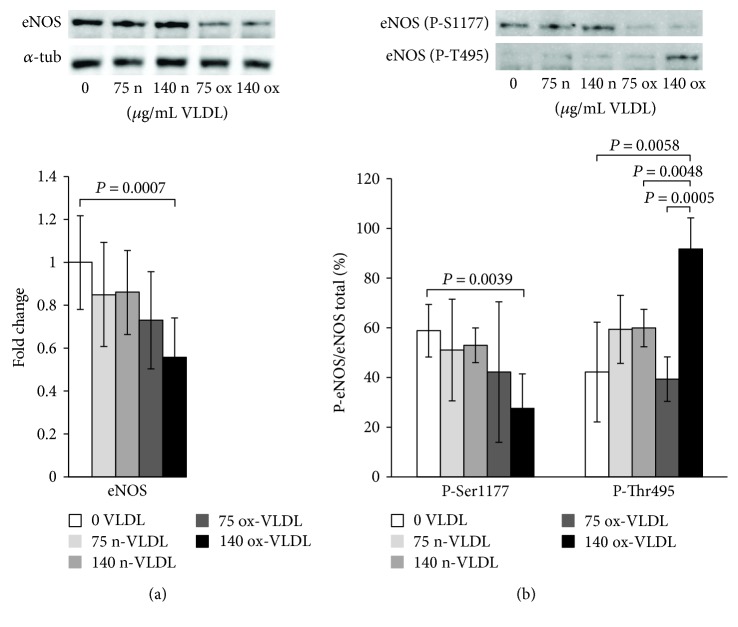
eNOS protein expression and evaluation of uncoupling in response to VLDL by Western blot. HUVEC were incubated 24 h with (75 and 140 *μ*g/mL) n-VLDL or ox-VLDL and then assayed by Western blot with anti-eNOS antibodies (see Materials and Methods). (a) Levels of eNOS protein after n-VLDL or ox-VLDL treatment. Densitometric values are shown as fold change versus the eNOS protein expressed by control cells. Data ± SD, *n*. of biological experiments = 8. *P* values were considered statistically significant by ANOVA. Inset: Western blot pattern of eNOS after 24 h incubation with n-VLDL or ox-VLDL; *α*-tubulin as reference (*α*-tub). (b) Phosphorylation of eNOS at Ser-1177 and Thr-495 in response to n-VLDL or ox-VLDL (see Materials and Methods). Data ± SD, *n*. of biological experiments = 4. *P* values were considered statistically significant by ANOVA. Inset: Western blot pattern of eNOS (P-S1177) and eNOS (P-T495) after 24 h incubation with n-VLDL or ox-VLDL.

The protein expression of eNOS was determined by Western blot analysis in HUVEC incubated with n-VLDL or ox-VLDL. For the analysis, primary antibodies against eNOS, or specifically recognising Thr495- or Ser1177-phosphorylated eNOS, were used. Consistent with the data shown in Figure 1, the exposure to n-VLDL leads to a ~20% decrease of the eNOS protein detected regardless to the lipoprotein concentration used, namely, 75 and 140 *μ*g mL^−1^. The exposure to ox-VLDL, at the same concentrations, induces, respectively, a ~30% and ~40% decrease of protein expression (Figure 2(a)).

The results of Western blot carried out with primary antibodies directed against phosphorylated eNOS indicated differences in phosphorylation at the level of key regulatory sites, Ser1177 and Thr495, upon varying VLDL redox state and concentration. In Figure 2(b), the results were reported as the ratio of phosphorylated eNOS (at Ser1177 or Thr495) over total eNOS (%). Control cells exhibited a ~60% phosphorylation at Ser1177 (P-Ser1177), and incubation with n-VLDL (both 75 and 140 *μ*g mL^−1^) did not induce significant changes. Nevertheless, the amount of P-Ser1177 decreased significantly to ~30%–40% of the total eNOS, when cells were incubated with ox-VLDL (Figure 2(b)). Somewhat symmetrically, Thr495 (P-Thr495) is ~40% phosphorylated in the controls, reaching ~60% phosphorylation upon incubation with n-VLDL, regardless to their concentration, in the limited range explored. At the highest ox-VLDL concentration, up to ~90% threonin was phosphorylated (Figure 2(b)).

### 3.5. Nitrite/Nitrate, Reactive Oxygen Species, and 3-Nitrotyrosine

**Figure 3:**
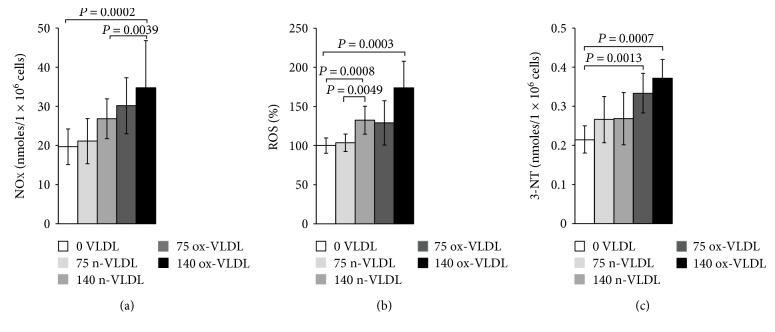
Oxidative and nitrosative stress driven in HUVEC by VLDL. Assays were carried out following 24 h incubation of HUVEC with n-VLDL or ox-VLDL (75 and 140 *μ*g/mL). (a) Nitrite-Nitrate (NOx) accumulation. NOx accumulation (24 h) was quantified in the cell supernatant downstream treatments by 2,3-diaminonaphthalene (DAN) as described in the Materials and Methods section. Data ± SD; *n*. of biological experiments = 5. *P* values were considered statistically significant by ANOVA. (b) Reactive oxygen species (ROS) production. Intracellular ROS levels were measured in VLDL-treated and VLDL-untreated EC in the presence of 2,7-dichlorodihydrofluorescein diacetate (DCFDA) (see Materials and Methods). The DCFDA fluorescence was followed kinetically and the values taken at 30 min. Data are shown as percentage of ROS amount detected in untreated cells and normalised for protein content. Data are the means ± SD; *n*. of biological experiments = 6. *P* values were considered statistically significant by ANOVA. (c) 3-Nitrotyrosine (3-NT) determination. The 3-NT content was measured by nitrotyrosine competitive ELISA (see Materials and Methods) in VLDL-treated and VLDL-untreated cells (lysate). Data values are the means ± SD; *n*. of biological experiments = 5. *P* values were considered statistically significant by ANOVA.

The accumulation of nitrite/nitrate (NOx), the production of ROS, and the concentration level of 3-NT were measured after 24 h incubation with VLDL. As shown in Figure 3(a), ~20 nmoles NOx was produced by 10^6^ control cells in 24 h. Incubation with VLDL resulted in the increase of NOx production, as a function of VLDL concentration and oxidation state. 75 *μ*g/mL and 140 *μ*g/mL ox-VLDL resulted in the highest NOx increase, respectively, by 1.5- and 2-fold with respect to controls. The production of ROS was also measured under similar conditions. As shown in Figure 3(b), the ROS level rises during the 24 h incubation, with increasing the concentration of VLDL, and more markedly if these are oxidised; the larger effect is observed at the highest ox-VLDL concentration. A comparable trend is observed when monitoring the level of 3-nitrotyrosine protein modification; this last finding, particularly, suggests a correlation between the persistence in the HUVEC environment of ox-VLDL and formation of the short-lived detrimental peroxynitrite (Figure 3(c)).

### 3.6. HUVEC Respiration

The O_2_ consumption of HUVEC following treatment with n-VLDL or ox-VLDL was measured oxigraphically. Measurements were carried out under different experimental conditions, such as using intact or digitonin-permeabilised cells in the presence or absence of specific mitochondrial substrates and respiratory chain inhibitors.

**Figure 4:**
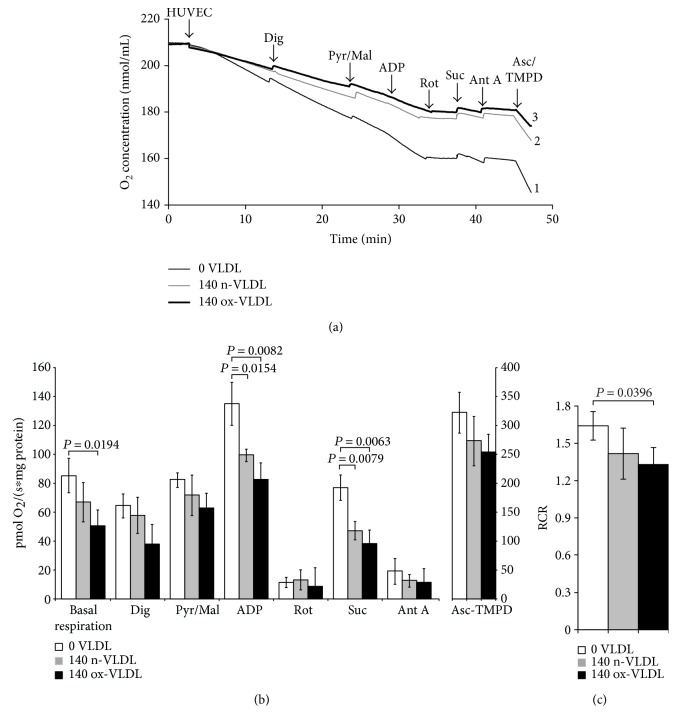
Evaluation of mitochondrial OXPHOS in response to VLDL treatment by O_2_ consumption measurements. (a) O_2_ consumption profiles. The oxygen consumption was monitored in 1 × 10^6^ HUVEC after 24 h incubation under the conditions indicated; thin line: control cells (1); grey line: 140 *μ*g mL^−1^ n-VLDL (2); bold line: 140 *μ*g mL^−1^ ox-VLDL (3). Digitonin (Dig) was used to permeabilise cells. Substrates/inhibitors added along the trace: pyruvate and malate (Pyr/Mal); adenosine diphosphate (ADP); rotenone (Rot); succinate (Suc); antimycin A (Ant A); ascorbate and N,N,N′,N′-tetrametil-p-fenilendiammina (Asc/TMPD). (b) O_2_ consumption rates. Respiration rate values measured in VLDL-treated HUVEC after each addition step (of substrate/inhibitor). Data values are the means ± SD; *n*. of biological experiments = 3. *P* values were considered statistically significant by ANOVA. (c) Respiratory control ratio (RCR). The values of RCR were obtained as the ratio between the O_2_ consumption rate measured in the presence of saturating [ADP] (state 3) and in its absence (state 4). Data values are the means ± SD; *n*. of biological experiments = 3. *P* values were considered statistically significant by ANOVA.

Typical traces are reported in Figure 4(a), where HUVEC untreated or incubated with n-VLDL or ox-VLDL, both 140 *μ*g mL^−1^, were allowed to consume O_2_ in the presence and absence of selective activators or inhibitors of specific respiratory chain complexes [49, 50]. The basal rates of O_2_ consumption have been measured and reported under all conditions (see Figure 4(b)). As shown in the figure, the incubation with n-VLDL induces a ~21% decrease of the O_2_ consumption, from ~85 pmoles O_2_ s^−1^ to ~67 pmoles O_2_ s^−1^. Moreover, the rate of respiration decreases, instead down to ~59% of the initial value, if incubation is carried out with ox-VLDL.

The depression of O_2_ consumption appears slightly more evident in the presence of rotenone and succinate, inhibiting complex I and activating complex II, respectively (Figure 4(b)). The basal respiratory control ratio, RCR, was also affected by the VLDL incubation; it decreased from 1.74 to 1.50 and 1.40, upon treatment with n-VLDL and ox-VLDL, respectively (see Figure 4(c)).

## 4. Discussion

The endothelium of vessels exposed to oxidative stress displays an altered availability of reactive oxygen and nitrogen species (RONS) that may result in disturbance of mitochondrial function and unbalancing of cell physiological signalling, aspects in which NO is known to play a crucial role [31, 51–54].

Bioavailability of NO at cell level depends on the activity of the NOS isoforms, so that physiological pathways ascribable to eNOS activity are featured by a limited (nM) amount of NO, whereas higher NO concentrations (*μ*M) resulting from the activation of iNOS are responsible for structural modifications such as membrane lipid peroxydation and protein nitrosation. These modifications are associated to the onset and maintenance of severe inflammatory states, including atherosclerosis [34, 55, 56].

In this study, human umbilical vein endothelial cells (HUVEC) have been used as a model system to investigate the endothelium response to a persistent VLDL exposure.

The incubation time and the VLDL concentrations were chosen so to set a mild though clear dyslipidemic condition of pathophysiological relevance. Within the experimental limits of cells in culture mimicking endothelium microenvironment, cells were allowed to face for 24 h about twice as much the physiological blood concentration of VLDL (140 *μ*g/mL), as prepared (native) or oxidized. The experimental design was such to investigate the role of VLDL in inducing nitro-oxidation of endothelium-like cells.

The analysis of NOS expression suggested the activation of a regulative antagonistic cross talk among the two NOS isoforms (eNOS and iNOS), so that the upregulation of the iNOS mRNA was accompanied, under comparable conditions, by a significant downregulation of the eNOS.

The iNOS activation is fully consistent with the increased production of NOx independently detected in the culture medium, suggesting a pathway of NO production which escapes the eNOS control. Furthermore, the increased production of ROS and 3-nitrotyrosine herein reported is consistent with the evidence of peroxynitrite formation compatible with a cogent inflammatory state of the cells [34].

The whole framework is also coherent with the upregulation of IL8 and VEGFA, genes involved in NO production and cell inflammatory response [57] and with the increased expression of NCF2 and NQO1 factors related to the NADPH oxidase function and biosynthesis [58, 59].

Consistently with the setting of an inflammatory state, the lowered eNOS synthesis induced by VLDL in HUVEC may imply an alteration of the physiological vasodilatory function relying on nanomolar NO fluxes. It is worth to notice that the eNOS is not only downregulated, according to both the mRNA and protein expression changes: based on the detected eNOS phosphorylation pattern (by targeting phospho-Ser1177 and phospho-Thr495), the enzyme appears also largely uncoupled hence supporting the production of superoxide ion [60–62].

The relevance of the proposed involvement of VLDL in the biosynthetic and functional regulation of eNOS was supported by changes found, over the same time scale, on the regulation of genes involved to NOS stability and function [63, 64]. The decreased expression of DDAH2, scavenger of an eNOS inhibitor (ADMA) [65], as well as the upregulation of ARG2, responsible for the depletion of arginine, were all elements pointing to the impairment of the physiological production of NO.

Interestingly, and somehow discordant with the above findings, some differences in the expression of other genes from the same array, as the modest upregulation of HSP90, involved in maintaining the dimeric structure of eNOS [66] and of GLA, responsible for degradation of the eNOS uncoupler Gb3 [67], seemed to point out the activation of compensatory pathways possibly aimed to the preservation of the eNOS function. Furthermore, the upregulation of the enzyme GCH1, involved in BH4 biosynthesis, combined to the downregulation of its feedback regulator GCHFR, suggests that under conditions in which BH4 oxidation is feasible, such as under oxidising conditions, signals enhancing BH4 bioavailability may be activated by endothelial cells, aimed at preventing or possibly reverting eNOS uncoupling [38, 68].

It is interesting to underline that upon VLDL treatment, the observed increase in ROS concentration follows a trend fully consistent with the progress of eNOS uncoupling, detected by Western blot. A peak in ROS concentration was found after 140 *μ*g/mL ox-VLDL administration, a condition matching that responsible for the ultimate eNOS uncoupling (lowest phospho-Ser1177, highest phospho-Thr495).

It may be also interesting to point out that the increased production of superoxide goes along with a mild upregulation of SOD1 and SOD2 mRNAs (Figure 1(d)), thus suggesting a cell attempt to tempering ROS rise.

Beside NO, ROS are also involved in the maintenance of physiological vascular homeostasis, through a tight control exerted by healthy endothelial cells.

The setting of prooxidative conditions was shown to result in increased ROS production, associated with the activation of signals promoting oxidative damage [22, 69].

Hence, the VLDL-induced cell redox changes are characterised by the concurrent synthesis of NO by the iNOS and of O_2_^−•^ by the uncoupled eNOS. These two evidences are fully compatible with the observed production of the highly reactive species peroxynitrite, probed by the increased tyrosine nitration.

Ox-VLDL treatment appears to be the condition necessary and sufficient to determine peroxynitrite formation, as the increase of 3-NT was found significant already at 75 *μ*g/mL, compared to control cells; this may suggest a specific interaction of ox-VLDL with the scavenger receptors. As already reported for ox-LDL, this class of cell surface receptors may specifically recognise oxidised lipoproteins, thus activating typical inflammatory cell response [70, 71].

The whole picture appears consistent with the onset and/or maintenance of a nitrosative stress in endothelial cells undergoing VLDL treatments; within this frame, the determination of the functional state of mitochondria is relevant to define the level of cell dysfunction.

VLDL induced a functional decrease (mild if native, more evident if oxidised) of mitochondrial respiration. The mitochondrial respiratory control ratio (RCR) also followed a similar trend. The respiratory chain activity, evaluated at the level of the specific mitochondrial complexes, showed a decrease of the electron transfer efficiency (~20%), detected almost uniformly along the chain, apparently just slightly more at the level of complex II (~50% inhibition by ox-VLDL).

As the observed impairment on mitochondrial respiration may not be unequivocally attributed to a specific respiratory complex, it appears feasible to envisage that the oxi/nitrosative stress induced by VLDL may determine relevant modification of mitochondrial proteins, such as protein nitrosation, causing an interference with the electron transfer process. The persistency of these conditions would bring to supercomplex disaggregation and alteration of the mitochondrial proteolipid arrangement.

## 5. Conclusions

From these experiments, we can propose that 24 h incubation of HUVEC with native and oxidised VLDL triggers a signalling leading to iNOS activation and eNOS uncoupling.

**Figure 5:**
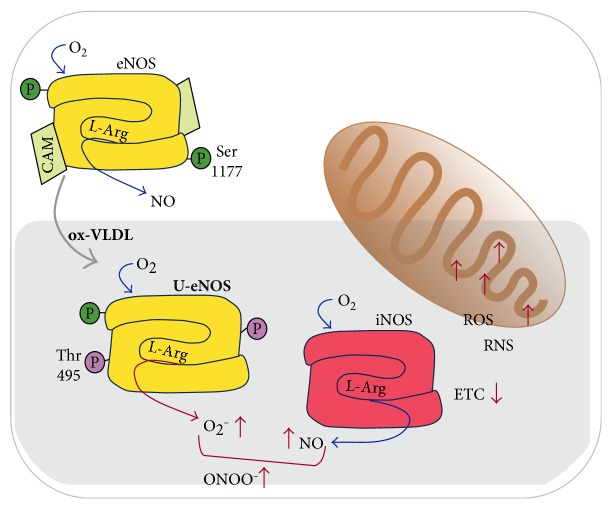
ox-VLDL-dependent EC dysfunction: schematic representation of the eNOS and iNOS involvement. The activity of eNOS (yellow) and iNOS (red) dimers are sketched. The coupled (native) eNOS is featured by the utilisation of substrates such as L-Arg and O_2_ and production of NO, in the presence of the cofactor calmodulin (CAM) and under condition of Ser1177 phosphorylation, promoting activity (white cell background). ox-VLDL triggers eNOS phosphorylation at residue Thr495, with dephosphorylation of Ser1177: under these conditions, the eNOS is uncoupled (U-eNOS) and results in the generation of O_2_^−•^. In parallel, high level of iNOS biosynthesis occurs, leading to increased ROS and RNS production and resulting in the down-modulation of mitochondrial function (gray background).

These events, resulting in an unbalance of NO and ROS metabolism, are more clearly produced by excess of ox-VLDL leading to production of harmful proxynitrite and triggering a cell inflammatory state. Consistently, cells display a lower mitochondrial O_2_ consumption and RCR (Figure 5).

It is tempting to speculate on the biomedical relevance of these results: an altered lipid metabolism arising from both a genetic or epigenetic background could represent a condition in which an inadequate lifestyle (smoking abuse, highly processed food consumption, and pollution) provides an additional detrimental contribution, perceived by the endothelium of vessel as a prooxidant insult.

According to our results, the early dysfunction of the eNOS associated to iNOS activation driven by VLDL can be relevant to set conditions compatible with the development of atherosclerosis.

## Supplementary Material

Supplementary Fig. 1 VLDL isolation and oxidation. a) Polyacrylamide gel electrophoresis (SDS-PAGE) analysis of n-VLDL and ox-VLDL. VLDL fractions (20 μg of protein) were subjected to SDS (0.1%)-gelelectrophoresis (4% to 12% gradient gel). Lane 1, molecular weight markers; lane 2, native VLDL (n-VLDL); lane 3, VLDL oxidised (ox-VLDL) 6 h at 37 °C with 40 μM CuSO4. b) Lipoprotein electrophoresis on agarose gel. VLDL fractions were running on 0.6% agarose gel and stained with Sudan Black B. Lane 1, serum blood; lane 2: n-VLDL fraction. c) The oxidation kinetics of VLDL by copper concentrations from 5 μM to 40 μM. VLDL oxidation kinetics was monitored by following the conjugated diene (CD) formation (monitoring the temporal change in absorbance at 234 nm). Concentrations of CD were calculated using ε234 ⁼ 29500 M^−1^cm^−1^. d) The thiobarbituric acid-reactive substances (TBARS) levels of VLDL incubated 6 h at 37 °C in the presence and absence of 5 μM and 40 μM CuSO_4_. The VLDL peroxidation was estimated by the method of Kosugi, Kojima, and Kikugawa. TBARS assay values are reported in malondialdehyde (MDA) equivalents. Data values are the means ± SD; n. of biological experiments ⁼ 5.

## Figures and Tables

**Table 1:** Expression level of genes involved in the NO signalling, superoxide metabolism, and oxidative stress response induced by n-VLDL and ox-VLDL in HUVEC.

Functional grouping	Gene	n-VLDL (rel. exp.)^#^	*P* value n-VLDL versus 0 VLDL	ox-VLDL (rel. exp.)^#^	*P* value ox-VLDL versus 0 VLDL	*P* value ox-VLDL versus n-VLDL
Nitric oxide metabolism	*NOS1 (nNOS)*	—	—	—	—	—
Nitric oxide biosynthesis	*NOS2 (iNOS)*	3521 ± 0.684	0.0005^∗^	15,212 ± 7287	0.0099^∗^	0.0513
	*NOS3 (eNOS)*	0.772 ± 0.031	0.0047^∗^	0.602 ± 0.173	0.0095^∗^	0.1857
Nitric oxide biosynthesis	*DYNLL1*	0.996 ± 0.450	0.9860	1066 ± 0.345	0.7615	0.8376
Regulation	*GLA*	0.921 ± 0.253	0.6263	1438 ± 0.231	0.0424	0.1046
	*HSP90AB1*	1157 ± 0.393	0.5550	1553 ± 0.343	0.0485	0.2456
	*IL8*	0.551 ± 0.507	0.0794	6532 ± 2814	0.0272^∗^	0.0654
	*INS*	—	—	—	—	—
	*NOS1AP*	1037 ± 0.684	0.1139	2028 ± 0.395	0.0075^∗^	0.0125^∗^
Other nitric oxide	*AKT1*	1014 ± 0.189	0.5801	1356 ± 0.513	0.3054	0.7300
Biosynthesis genes	*ARG2*	1196 ± 0.265	0.3450	1847 ± 0.109	0.0006^∗^	0.0278
	*DDAH2*	1158 ± 0.281	0.4354	0.621 ± 0.386	0.0616	0.0439
	*EGFR*	2750 ± 2317	0.1798	2639 ± 1704	0.1045	0.9498
	*GCH1*	0.786 ± 0.119	0.0579	1354 ± 0.701	0.4329	0.2406
	*GCHFR*	0.685 ± 0.281	0.1486	0.327 ± 0.249	0.0136^∗^	0.1734
Nitric oxide induced	*CDKN1A*	1611 ± 1425	0.4282	1533 ± 0.904	0.3065	0.9399
	*CXCL8*	—	—	—	—	—
	*JUN*	1574 ± 0.655	0.2130	21,111 ± 0.890	0.0949	0.4297
	*VEGFA*	1451 ± 0.620	0.2582	5372 ± 1224	0.0134^∗^	0.0226^∗^
Nitric oxide suppressed	*CCNA1*	1335 ± 0.558	0.4048	1534 ± 0.128	0.0097^∗^	0.8953
	*MYB*	—	—	—	—	—
	*TROAP*	2080 ± 0.589	0.0192^∗^	1189 ± 0.156	0.1658	0.1917
Nitric oxide signalling	*CAMK1*	1477 ± 0.388	0.2150	1916 ± 0.473	0.0161^∗^	0.5063
	*DLG4*	1505 ± 0.935	0.3199	1178 ± 0.920	0.7098	0.6873
	*GRIN2D*	1461 ± 0.573	0.0832	1012 ± 0.554	0.9659	0.5407
	*PPP3CA*	1003 ± 0.388	0.4840	1386 ± 0.067	0.0155^∗^	0.9211
	*PRKAR1B*	1627 ± 0.620	0.1029	1472 ± 0.137	0.0259^∗^	0.7621
	*PRKCA*	1083 ± 0.036	0.5305	1909 ± 0.149	0.0019^∗^	0.0052^∗^
	*NQO1*	3456 ± 0.606	0.0018^∗^	2525 ± 0.835	0.0312^∗^	0.1657
Superoxide metabolism	*ALOX12*	1425 ± 0.450	0.0911	3253 ± 0.187	0.0001^∗^	0.0017^∗^
Release	*DUOX1*	1105 ± 0.317	0.1697	0.404 ± 0.048	0.0029^∗^	0.0115^∗^
Oxidoreductases	*DUOX2*	1429 ± 0.949	0.3574	0.439 ± 0.069	0.0001^∗^	0.1435
Peroxidases	*NOX5*	—	—	—	—	—
	*PRG3*	—	—	—	—	—
	*SOD1*	0.897 ± 0.235	0.4863	1608 ± 0.492	0.1094	0.1532
	*SOD2*	0.942 ± 0.414	0.8203	1819 ± 0.502	0.0539	0.1200
	*SOD3*	—	—	—	—	—
Other superoxide	*CCS*	1296 ± 0.670	0.4132	1173 ± 0.518	0.5367	0.8134
Metabolism genes	*NCF1*	—	—	—	—	—
	*NCF2*	1250 ± 0.698	0.4988	3637 ± 1943	0.0549	0.1460
	*PREX1*	0.916 ± 0.191	0.6021	0.857 ± 0.255	0.4455	0.7658
Oxidative stress	*MPO*	—	—	—	—	—
Antiapoptotic	*MTL5*	0.756 ± 0.316	0.1880	0.759 ± 0.479	0.3562	0.9919
	*NME5*	1049 ± 0.249	0.7349	1645 ± 0.939	0.2187	0.3483
	*PRDX2*	0.999 ± 0.294	0.9952	1040 ± 0.029	0.7286	0.8198
	*RNF7*	1260 ± 0.655	0.5334	1248 ± 0.140	0.0863	0.9827
Antioxidants	*APOE*	—	—	—	—	—
	*MT3*	—	—	—	—	—
	*VIMP*	—	—	—	—	—
	*SRXN1*	2721 ± 2766	0.2554	8175 ± 3135	0.0075^∗^	0.1113
Glutathione peroxidases	*GPX1*	1355 ± 0.587	0.4132	1070 ± 0.387	0.8239	0.5210
	*GPX2*	1392 ± 1035	0.4698	0.890 ± 0.314	0.5164	0.4669
	*GPX3*	1639 ± 0.795	0.1599	1683 ± 0.682	0.0960	0.9458
	*GPX4*	1436 ± 0.894	0.4714	1283 ± 0.663	0.5417	0.8239
	*GPX5*	—	—	—	—	—
	*GPX6*	—	—	—	—	—
Other oxidoreductases	*CAT*	1079 ± 0.533	0.8239	1287 ± 0.542	0.4419	0.6603
	*EPX*	1132 ± 0.448	0.4422	2491 ± 0.655	0.0087^∗^	0.1634
	*LPO*	—	—	—	—	—
	*MSRA*	1298 ± 0.793	0.48110	1062 ± 0.458	0.8025	0.6788
	*PRDX6*	1918 ± 1369	0.3161	1526 ± 0.741	0.2989	0.6865
	*TPO*	0.802 ± 0.688	0.5814	0.868 ± 0.095	0.1235	0.9056
	*TXNRD2*	1746 ± 0.944	0.1712	0.933 ± 0.284	0.6938	0.2250
Other peroxidases	*CSDE1*	1532 ± 1115	0.4558	0.868 ± 0.558	0.7056	0.4081
	*CYGB*	—	—	—	—	—
	*GPR156*	—	—	—	—	—
	*PRDX2*	0.999 ± 0.294	0.9953	1040 ± 0.029	0.7286	0.8198
	*PRDX5*	1412 ± 0.845	0.3940	1035 ± 0.537	0.9149	0.5503
	*TTN*	0.416 ± 0.095	0.0008^∗^	0.350 ± 0.192	0.0026^∗^	0.7083
Regulation	*FOXM1*	1541 ± 0.369	0.0670	1695 ± 0.914	0.2597	0.8012
	*GLRX2*	1006 ± 0.447	0.9657	1323 ± 0.778	0.5124	0.6323
	*SCRT2*	—	—	—	—	—
	*SIRT2*	1322 ± 0.502	0.3329	1339 ± 0.292	0.1279	0.9676
Other oxidative stress	*ATOX1*	1254 ± 0.913	0.6551	1515 ± 0.757	0.3056	0.7224
Response genes	*DUSP1*	1186 ± 0.189	0.3110	1490 ± 0.797	0.1381	0.3571
	*GSS*	1357 ± 0.684	0.4227	1124 ± 0.173	0.3452	0.5988
	*KRT1*	—	—	—	—	—
	*MBL2*	2039 ± 1129	0.1870	16,376 ± 10,431	0.1108	0.1335
	*NUDT1*	1521 ± 1018	0.3459	1871 ± 0.589	0.0340	0.6342
	*OXR1*	0.876 ± 0.199	0.3577	1044 ± 0.606	0.8910	0.6727
	*PNKP*	1382 ± 0.601	0.2771	1566 ± 0.907	0.2675	0.7837
	*PRNP*	2908 ± 2329	0.2226	5331 ± 2944	0.0284	0.4218
	*SCARA3*	0.762 ± 0.376	0.3352	0.482 ± 0.277	0.0318	0.3568
	*SEPP1*	—	—	—	—	—
	*SGK2*	4371 ± 5854	0.3548	8491 ± 5139	0.0698	0.5327

n-VLDL = RT-PCR assay performed after 24 h incubation of HUVEC with native VLDL (140 *μ*g/mL); ox-VLDL = RT-PCR assay performed after 24 h incubation of HUVEC with oxidised VLDL (140 *μ*g/mL). ^#^Relative expression (rel. exp.) versus untreated cells (0 VLDL) grown for an overall comparable time. cDNAs undetectable under the condition assayed were indicated by “—.” *P* values considered statistically significant by ANOVA were marked by “^∗^.”

## Abbreviations

ADMA: Asymmetric dimethylarginine
ADP: Adenosine diphosphate
ALOX12: Arachidonate 12-lipoxygenase, 12S type
ARG2: Arginase, type II
BSA: Bovine serum albumin
cDNA: Complementary DNA
DDAH2: Dimethylarginine dimethylaminohydrolase 2
EC: Endothelial cells
eNOS: Endothelial nitric oxide synthase
EDTA: Ethylenediaminetetraacetic acid
Gb3: Globotriaosylceramide
GCH1: GTP cyclohydrolase 1
GCHFR: GTP cyclohydrolase I feedback regulator
GLA: Galactosidase alpha
HSP90AB1: Heat shock protein 90 kDa *α*, class B member 1
HUVEC: Human umbilical vein endothelial cells
IL8: Interleukin 8
iNOS: Inducible NOS
LDL: Low-density lipoproteins
NCF2: Neutrophil cytosolic factor 2
NO: Nitric oxide
nNOS: Neuronal NOS
NOx: Nitrites and nitrates
NQO1: NAD(P)H dehydrogenase, quinone 1
n-VLDL: Native VLDL
ox-VLDL: Oxidised VLDL
PBS: Phosphate-buffered saline
PCR: Polymerase chain reaction
P-Ser1177: Phosphorylated serine 1177
P-Thr495: Phosphorylated threonine 495
RNS: Reactive nitrogen species
ROS: Reactive oxygen species
RONS: Reactive oxygen and nitrogen species
Thr: Threonine
RCR: Respiratory control ratio
Ser: Serine
SOD1: Superoxide dismutase 1
SOD2: Superoxide dismutase 2
VEGFA: Vascular endothelial growth factor A
VLDL: Very-low-density lipoproteins
U-eNOS: Uncoupled eNOS
3-NT: 3-Nitrotyrosine.
