# Supplementary material for: VLDL Induced Modulation of Nitric Oxide Signalling and Cell Redox Homeostasis in HUVEC
Source: Oxid Med Cell Longev. 2017 Sep 20;2017:2697364. doi: 10.1155/2017/2697364 (PMC5632467; doi:10.1155/2017/2697364)
Supplement: Supplementary file 1 — Supplementary Fig. 1 VLDL isolation and oxidation. a) Polyacrylamide gel electrophoresis (SDS-PAGE) analysis of n-VLDL and ox-VLDL. VLDL fractions (20 μg of protein) were subjected to SDS (0.1%)-gelelectrophoresis (4% to 12% gradient gel). Lane 1, molecular weight markers; lane 2, native VLDL (n-VLDL); lane 3, VLDL oxidised (ox-VLDL) 6 h at 37 °C with 40 μM CuSO4. b) Lipoprotein electrophoresis on agarose gel. VLDL fractions were running on 0.6% agarose gel and stained with Sudan Black B. Lane 1, serum blood; lane 2: n-VLDL fraction. c) The oxidation kinetics of VLDL by copper concentrations from 5 μM to 40 μM. VLDL oxidation kinetics was monitored by following the conjugated diene (CD) formation (monitoring the temporal change in absorbance at 234 nm). Concentrations of CD were calculated using ε234 ⁼ 29500 M−1cm−1. d) The thiobarbituric acid-reactive substances (TBARS) levels of VLDL incubated 6 h at 37 °C in the presence and absence of 5 μM and 40 μM CuSO4. The VLDL peroxidation was estimated by the method of Kosugi, Kojima, and Kikugawa. TBARS assay values are reported in malondialdehyde (MDA) equivalents. Data values are the means ± SD; n. of biological experiments ⁼ 5. [file 2697364.f1.pdf]

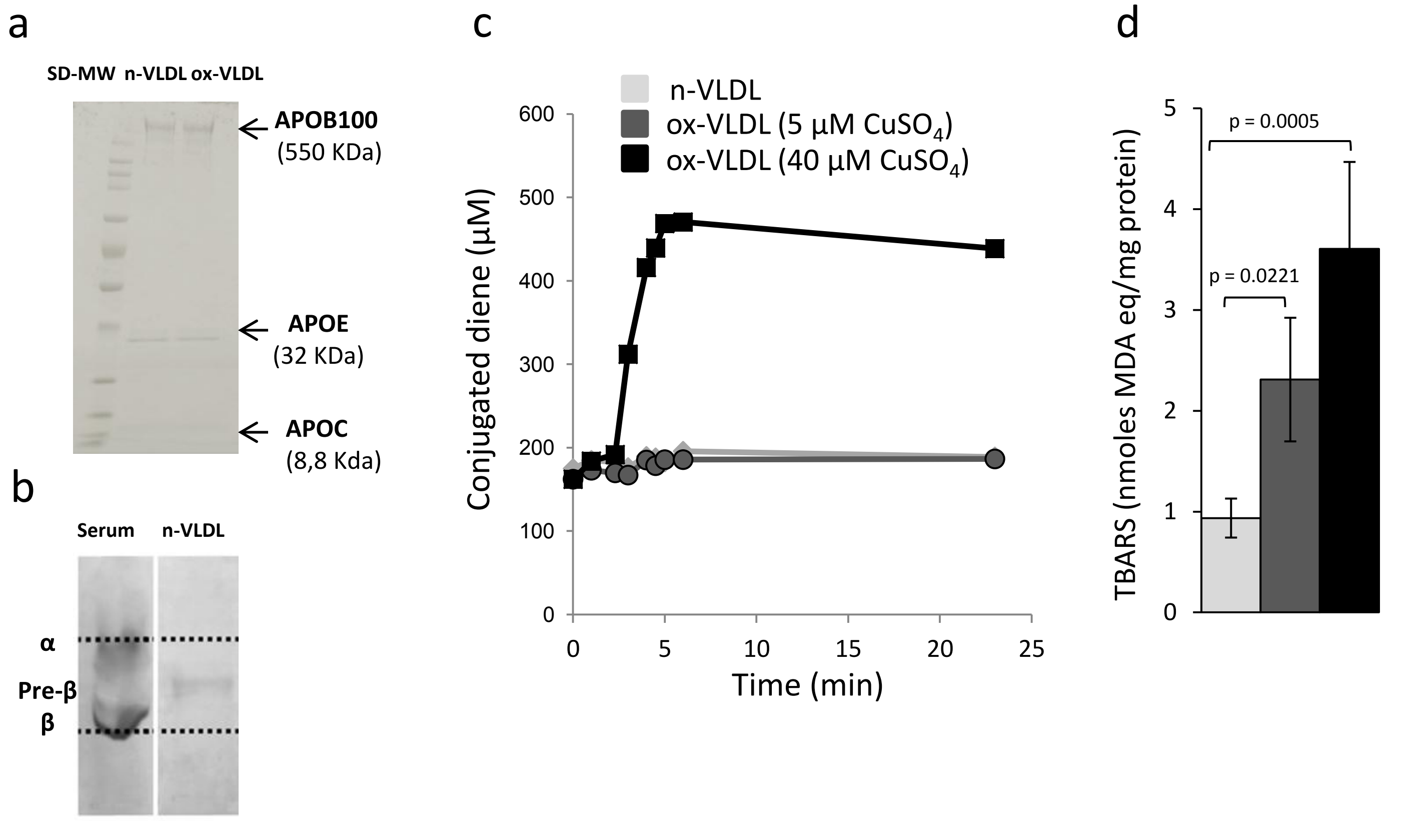

Supplementary Fig. 1

VLDL isolation and oxidation

a) Polyacrylamide gel electrophoresis (SDS-PAGE) analysis of n-VLDL and ox-VLDL. VLDL fractions (20 µg of protein) were subjected to SDS (0.1%)-gel electrophoresis (4% to 12% gradient gel). Lane 1, molecular weight markers; lane 2, native VLDL (n-VLDL); lane 3, VLDL oxidised (ox-VLDL) 6 h at 37 °C with 40 µM $CuSO_4$. b) Lipoprotein electrophoresis on agarose gel. VLDL fractions were running on 0.6% agarose gel and stained with Sudan Black B. Lane 1, serum blood; lane 2: n-VLDL fraction. c) The oxidation kinetics of VLDL by copper concentrations from 5 µM to 40 µM. VLDL oxidation kinetics was monitored by following the conjugated diene (CD) formation (monitoring the temporal change in absorbance at 234 nm). Concentrations of CD were calculated using ε234 = 29500 $M^{-1}cm^{-1}$. d) The thiobarbituric acid-reactive substances (TBARS) levels of VLDL incubated 6 h at 37 °C in the presence and absence of 5 µM and 40 µM $CuSO_4$. The VLDL peroxidation was estimated by the method of Kosugi, Kojima, and Kikugawa. TBARS assay values are reported in malondialdehyde (MDA) equivalents. Data values are the means ± SD; n. of biological experiments = 5.
